# The role of previously unmeasured organic acids in the pathogenesis of severe malaria

**DOI:** 10.1186/s13054-015-1023-5

**Published:** 2015-09-07

**Authors:** M. Trent Herdman, Natthida Sriboonvorakul, Stije J. Leopold, Sam Douthwaite, Sanjib Mohanty, M. Mahtab Uddin Hassan, Richard J. Maude, Hugh WF Kingston, Katherine Plewes, Prakaykaew Charunwatthana, Kamolrat Silamut, Charles J. Woodrow, Kesinee Chotinavich, Md. Amir Hossain, M. Abul Faiz, Saroj Mishra, Natchanun Leepipatpiboon, Nicholas J. White, Nicholas PJ Day, Joel Tarning, Arjen M. Dondorp

**Affiliations:** Mahidol-Oxford Tropical Medicine Research Unit, Faculty of Tropical Medicine, Mahidol University, Bangkok, Thailand; Department of Chemistry, Faculty of Science, Chulalongkorn University, Bangkok, Thailand; Department of Clinical Tropical Medicine, Faculty of Tropical Medicine, Mahidol University, Bangkok, Thailand; Ispat General Hospital, Rourkela, Orissa India; Chittagong Medical College Hospital, Chittagong, Bangladesh; Centre for Tropical Medicine and Global Health, Nuffield Department of Medicine, University of Oxford, Oxford, UK; Global Health Division, Menzies School of Health Research, Darwin, Australia; Dev Care Foundation, Dhaka, Bangladesh

## Abstract

**Introduction:**

Severe falciparum malaria is commonly complicated by metabolic acidosis. Together with lactic acid (LA), other previously unmeasured acids have been implicated in the pathogenesis of falciparum malaria.

**Methods:**

In this prospective study, we characterised organic acids in adults with severe falciparum malaria in India and Bangladesh. Liquid chromatography-mass spectrometry was used to measure organic acids in plasma and urine. Patients were followed until recovery or death.

**Results:**

Patients with severe malaria (n=138), uncomplicated malaria (n=102), sepsis (n=32) and febrile encephalopathy (n=35) were included. Strong ion gap (mean±SD) was elevated in severe malaria (8.2 mEq/L±4.5) and severe sepsis (8.6 mEq/L±7.7) compared with uncomplicated malaria (6.0 mEq/L±5.1) and encephalopathy (6.6 mEq/L±4.7). Compared with uncomplicated malaria, severe malaria was characterised by elevated plasma LA, hydroxyphenyllactic acid (HPLA), α-hydroxybutyric acid and β-hydroxybutyric acid (all *P*<0.05). In urine, concentrations of methylmalonic, ethylmalonic and α-ketoglutaric acids were also elevated. Multivariate logistic regression showed that plasma HPLA was a strong independent predictor of death (odds ratio [OR] 3.5, 95 % confidence interval [CI] 1.6–7.5, *P*=0.001), comparable to LA (OR 3.5, 95 % CI 1.5–7.8, *P*=0.003) (combined area under the receiver operating characteristic curve 0.81).

**Conclusions:**

Newly identified acids, in addition to LA, are elevated in patients with severe malaria and are highly predictive of fatal outcome. Further characterisation of their sources and metabolic pathways is now needed.

**Electronic supplementary material:**

The online version of this article (doi:10.1186/s13054-015-1023-5) contains supplementary material, which is available to authorized users.

## Introduction

Severe falciparum malaria remains a leading cause of mortality in the tropics. The case fatality rate in adult patients remains between 15 % and 40 % in resource-constrained hospital settings, and it is 11 % in intensive care units in resource-rich settings [1–3]. A better understanding of the pathophysiology of severe malaria is necessary to guide the development of new therapies.

The degree of metabolic acidosis in severe malaria is a strong prognosticator for a fatal outcome. Acidosis is associated with hyperlactataemia, and lactic acid (LA) has therefore been considered a major contributor to hydrogen ion accumulation [4, 5]. In contrast to bacterial sepsis, shock is not a common manifestation of severe malaria. Profound hyperlactataemia can occur in the presence of apparently normal macrovascular circulation [6, 7]. In severe falciparum malaria, sequestration of parasitised erythrocytes in capillaries and venules impairs oxygen delivery, leading to anaerobic glycolysis and consequent lactic acidosis with an increased lactate/pyruvate ratio [4, 8–10]. Further compromise of the microcirculatory flow occurs as a result of reduced erythrocyte deformability, rosette formation, agglutination, of parasitised erythrocytes, severe anaemia and endothelial dysfunction [4, 11, 12]. Compounding increased lactate production, clearance of lactate may be reduced owing to impaired gluconeogenesis and decreased liver blood flow [4, 13]. Renal dysfunction also contributes to acidosis through loss of the kidney’s normal role in acid–base homeostasis [14]. Yet, hyperlactataemia and renal dysfunction together still account for only two-thirds of the variance in standard base deficit (SBD) [4].

Using the modified physicochemical approach of Stewart, the presence of an unmeasured anion load was detected in patients with severe malaria [15, 16]. The mean (95 % confidence interval [CI]) strong ion gap (SIG), representing unmeasured acids, was 11.1 (10.4–11.8) mEq/L (normal value 0–2 mEq/L), whilst the mean concentration of lactate was only 2.9 mmol/L (2.7–3.2). The SIG showed an independent correlation with mortality [16]. This suggests that, in addition to LA accumulation, other organic acids play an important role in the pathogenesis of severe malaria. The prognostic value and pathological importance of such organic acids has also been recognised in other critical illnesses, including bacterial sepsis [17–19]. Understanding of the pathophysiology of severe malaria can be enhanced by profiling hitherto unidentified acids and comparing this profile with the profiles of other disease states.

Using a recently developed liquid chromatography-mass spectrometry (LC-MS) assay [20], we quantified a range of additional acids in the plasma and urine of adults with severe malaria to assess these acids’ contributions to acid load and their prognostic significance. Reference groups of uncomplicated falciparum malaria, bacterial sepsis (as an alternative cause of metabolic acidosis) and non-malarial encephalopathy (as an alternative cause of fever and coma) were also characterised.

## Methods

### Site selection and participants

This observational study was conducted in Chittagong Medical College Hospital, Chittagong, Bangladesh, and Ispat General Hospital, Rourkela, India, from May 2009 to September 2012. Both are areas of low, seasonal malaria transmission. Non-pregnant adults older than 16 years of age were recruited into this prospective study. There were four clinical diagnostic categories: (1) uncomplicated falciparum malaria, (2) severe falciparum malaria, (3) sepsis (in the absence of malaria) and (4) non-malarial febrile encephalopathy. Malaria was defined by the presence of asexual *Plasmodium falciparum* parasites visualised on a peripheral blood film, and severe malaria was distinguished from uncomplicated malaria by the presence of one or more markers of severity according to modified World Health Organisation (WHO) criteria as defined by Tran et al. [21]. Sepsis was defined by a blood film negative for malaria of all species, clinical suspicion of bacterial infection (based on history, examination and, when available, complete blood count and other investigations), SBD ≥3 mEq/L and two or more of the following four criteria: (1) heart rate >90 beats per minute, (2) respiratory rate >20 breaths per minute, (3) systolic blood pressure <90 mmHg and/or (4) Glasgow Coma Scale (GCS) score <15. Encephalopathy was defined by a blood film negative for malaria of all species, documented fever (>37.5 °C), a GCS score <11 and onset of symptoms within 14 days of admission without an obvious non-infective aetiology (such as diabetic ketoacidosis or poisoning). Participants meeting the inclusion criteria for both sepsis and encephalopathy were classified as having sepsis. Participants with chronic kidney or liver disease were excluded.

Fully informed consent was obtained from the patient or a legally acceptable representative. The Chittagong Medical College Hospital Ethical Review Committee, the Ispat General Hospital Institutional Ethical Committee and the Oxford Tropical Research Ethics Committee approved this study.

### Study procedures

A detailed medical history and physical examination were performed upon enrolment, and each patient was monitored until discharge from the hospital or death. Anti-malarial medications and supportive therapies were administered in accordance with the WHO’s 2006 and 2010 malaria treatment guidelines and local hospital guidelines, although the availability of renal replacement therapy and mechanical ventilation was limited. Participants with sepsis or encephalopathy were treated according to hospital guidelines. Fluid management was carried out according to the attending physician’s discretion and typically entailed crystalloid administration guided by non-invasive monitoring. Upon patient admission, venous blood was collected for immediate processing in the hospital laboratory. Glucose, sodium, potassium, chloride, pH and partial pressure of carbon dioxide (pCO_2_) were assessed with an iSTAT point-of-care analyser (Abbott Point of Care, Maidenhead, UK). Bicarbonate (HCO_3_^−^) and SBD were derived internally from measurements of pH and pCO_2_ by using the iSTAT software. Haemoglobin and parasite count were determined using samples anti-coagulated with ethylenediaminetetraacetic acid (EDTA). Magnesium, calcium, phosphate, albumin, creatinine and total bilirubin were measured using freeze-thawed stored serum samples. l-Lactate was measured in plasma stored at −80 °C with fluoride/oxalate using the Olympus OSR 6193 system reagents for enzymatic quantification (Olympus Life Science, Southend-on-Sea, UK). Plasma *Plasmodium falciparum* histidine-rich protein 2 (*Pf*HRP2) concentrations were assessed from freeze-thawed plasma stored with EDTA using a commercial sandwich enzyme-linked immunosorbent assay kit (Cellabs, Brookvale, Australia) as described previously [9]. Urine for assessment of acids was collected either as a catheter specimen or as a fresh midstream sample.

### Liquid chromatography-mass spectroscopy of plasma and urine

Heparinised venous blood for LC-MS was centrifuged at 4 °C and 1500×*g* for 5 minutes, and plasma was stored at −80 °C or in liquid nitrogen until analysis. Unprocessed urine was stored in the same manner. The novel analytical techniques employed are described in detail elsewhere [20]. This technique permits simultaneous assessment of eight organic acids: LA, malonic acid (MA), methylmalonic acid (MMA), α-hydroxybutyric acid (α-HBA), β-hydroxybutyric acid (β-HBA), hydroxyphenyllactic acid (HPLA), ethylmalonic acid (EMA), and α-ketoglutaric acid (α-KGA). Urine concentrations were corrected for renal function by using the formula [A]_urine-corrected_ = [A]_urine_ × [Cre]_plasma_/[Cre]_urine_, as described previously for other urine substrates [22].

### Calculation of the strong ion gap and unmeasured anions

The apparent strong ion difference (SID_app_) was calculated as SID_app_ = [Na^+^] + [K^+^] + [Ca^2+^] + [Mg^2+^] − [Cl^−^] − [lactate^−^], with all concentrations expressed in milliequivalents per litre. The effective strong ion difference (SID_eff_) was calculated by inclusion of the charges contributed by HCO_3_^−^, albumin and inorganic phosphate, using calculations described previously [16]. SIG was calculated as SID_app_ − SID_eff_ as a measure of the remaining balance of charges contributed by unmeasured strong ions, including organic acids, and dissociated weak acids. For consistency with previous reports of the pathogenesis of acidosis in severe malaria, the SIG was calculated incorporating l-lactate derived by enzymatic assay.

### Statistical analysis

Statistical analysis was performed using STATA/IC 12.0 software (StataCorp, College Station, TX, USA) and Prism 6.0b software (GraphPad Software, La Jolla, CA, USA). Normally distributed continuous variables and log-transformed variables were compared between groups using analysis of variance, with post-test pairwise comparisons as appropriate, using Tukey’s multiple-comparisons test. Non-normally distributed data were compared using the Mann–Whitney *U* test. Associations between organic acids, clinical indicators and mortality caused by severe malaria were formally explored by calculation of Spearman’s rank correlation coefficient. For potential predictors of fatal outcome, odds ratios (ORs) and *P* values were determined by univariate logistic regression. Inclusion of explanatory variables in a multivariate model was hypothesis-driven. Two multivariate logistic regression models were designed, one including the four detectable plasma acids and one including the seven detectable urinary acids. Both models were assessed for collinearity using the ‘collin’ function in STATA/IC. Stepwise backward selection with a *P*-value cutoff of <0.05 was done to come to a final multivariate logistic regression model. For the final models, the area under the receiver operating characteristic curve (AUROCC) was determined, and goodness-of-fit was measured using the Hosmer-Lemeshow test and determination of the model’s *P* value.

## Results

### Baseline characteristics of the study population

A total of 138 consecutive patients with severe malaria and 102 with uncomplicated malaria were recruited to participate in this study. Non-malarial reference groups of acutely ill patients included 32 consecutively recruited participants with sepsis and 35 with febrile encephalopathy. At the time of admission, 63 % of patients with severe malaria were comatose, 46 % had jaundice, 17 % were severely anaemic, 16 % had acute kidney injury, 2 % were hypoglycaemic and 1 % had haemodynamic shock. Mortality rates were 28 % among participants with severe malaria, 50 % for those with sepsis and 43 % for patients with encephalopathy. The clinical and biochemical characteristics of these participants at the time of enrolment are shown in Table 1.

**Table 1:** Baseline clinical and biochemical characteristics of participants at the time of enrolment

Characteristics	Uncomplicated malaria N=102		Severe malaria N=138		Sepsis N=32		Encephalopathy N=35		*P* value*
Male, n (%)	80	(78 %)	97	(70 %)	15	(47 %)	23	(66 %)	0.008
Age, yr, mean (SD, range)	33	(14.8, 16–70)	36	(14.2, 16–75)	37	(14.7, 16–70)	40	(18.4, 18–75)	0.106
Respiratory rate, breaths/min, mean (SD, range)	26	(6.6, 18–48)	32	(10.2, 15–93)	34	(9.9, 15–58)	30	(9.4, 12–60)	<0.001
Heart rate, beats/min, mean (SD, range)	95	(16.2, 64–149)	110	(22.5, 54–166)	120	(16.9, 92–151)	104	(24.7, 60–170)	<0.001
Mean arterial pressure, mmHg, mean (SD, range)	81	(11.7, 57–115)	83	(15.2, 28–144)	79	(19.3, 20–127)	93	(17.1, 64–125)	<0.001
Shock, SBP <80 mmHg, n (%)	0	(0 %)	2	(1.4 %)	6	(19 %)	0	(0 %)	
Glasgow Coma Scale score, geo mean (95 % CI)	15	(15–15)	9	(9–10)	11	(9–13)	7	(6–8)	<0.001
GCS score <15, n (%)	15/102	(15 %)	109/138	(79 %)	17/32	(53 %)	35/35	(100 %)	
GCS score <11, n (%)	0	(0 %)	87/138	(63 %)	10/32	(31 %)	35/35	(100 %)	
GCS score <8, n (%)	0	(0 %)	28/138	(20 %)	7/32	(22 %)	10/35	(29 %)	
Parasitaemia (count/μl), geo mean (95 % CI)	11,184	(6853–18,253)	27,933	(18,101–43,105)					0.007
*Pf*HRP2 (ng/ml), geo mean (95 % CI)^a^	274	(201–376)	1,684	(1,323–2,143)					<0.001
Total bilirubin (mg/dl), geo mean (95 % CI, range)	1.1	(0.9–1.4, 0.2–28.3)	2.8	(2.3–3.5, 0.2–44.2)	0.94	(0.6–1.5, 0.2–10.9)	0.47	(0.3–0.6, 0.1–6.0)	<0.001
Creatinine (mg/dl), geo mean (95 % CI, range)	1.0	(0.9–1.0, 0.5–2.3)	1.5	(1.3–1.7, 0.5–8.6)	1.7	(1.2–2.3,0.3–14.1)	1.1	(0.9–1.4, 0.6–24.3)	<0.001
Acute kidney injury (creatinine >3 mg/dl)	0/101	(0 %)	20/135	(15 %)	6/31	(19 %)	2/35	(6 %)	
Hb (g/dl), mean (SD, range)	10.6	(2.8, 3.8–15.6)	9.6	(3.1, 2.6–17.5)	10.3	(3.0, 5.0–16.7)	12.3	(2.5, 4.7–16.1)	<0.001
Anaemia (Hb <7 g/dl), n (%)	13	(13 %)	24	(17 %)	3	(9 %)	2	(6 %)	
Glucose (mg/dl), geo mean (95 % CI, range)	122	(114–130, 55–332)	124	(115–134, 33–455)	126	(104–152, 39–443)	146	(124–171, 63–612)	0.205
Hypoglycaemia (<40 mg/dl), n (%)	0	(0 %)	3	(2 %)	1	(3 %)	0	(0 %)	
Mortality, n (%)	0	(0 %)	39	(28 %)	16	(50 %)	15	(43 %)	<0.001

*Abbreviations: GCS* Glasgow Coma Scale, *geo mean* geometric mean, *Hb* haemoglobin, *PfHRP2 Plasmodium falciparum* histidine-rich protein 2, *SBP* systolic blood pressure, *SD* standard deviation

*P values were derived by analysis of variance (ANOVA) between four participant groups (one-way ANOVA for normally distributed continuous variables or variables log-transformed toward normality; Kruskal-Wallis test for non-normally distributed variables). Student’s *t* tests were used for comparison of (log-transformed) parasite count and *Pf*HRP2 concentrations, where only two participant groups are compared. χ^2^ tests were used for comparison of mortality

^a^Owing to limited assay availability for *Pf*HRP2 analysis, data represent 95 participants for uncomplicated malaria and 126 for severe malaria

### Metabolic acidosis and the strong ion gap

The majority of participants with severe malaria had metabolic acidosis (mean SBD 6 mEq/L) (Table 2), but only 14 % of patients with severe malaria had a venous pH <7.35 compared with 47 % of patients with sepsis. SBD was different between groups (*P*<0.001), with higher mean SBD in patients with sepsis than in those severe malaria and higher SBD in patients with severe malaria than in patients with uncomplicated malaria or encephalopathy. SIG differed between study groups (*P*=0.002) and was higher in patients with severe malaria, sepsis or encephalopathy than in patients with uncomplicated malaria.

**Table 2:** Biochemical characteristics pertaining to acidosis and calculation of strong ion gap using admission venous blood specimens

Characteristics	Uncomplicated malaria N=102		Severe malaria N=138		Sepsis N=32		Encephalopathy N=35		*P* value*
Na^+^ (mmol/L)	134	(4.73, 109–143)	136	(6.90, 108–160)	136	(11.23, 107–172)	136	(9.18, 113–157)	0.163
K^+^ (mmol/L)	3.6	(0.44, 2.4–5.4)	3.9	(0.81, 2.1–7.1)	4.0	(1.24, 2.0–6.9)	3.7	(0.74, 2.0–5.5)	0.003
Mg^2+^ (mmol/L)	0.7	(0.22, 0.1–1.1)	0.9	(0.21, 0.5–1.8)	0.8	(0.22, 0.3–1.3)	0.8	(0.13, 0.4–1.1)	<0.001
Ca^2+^ (mmol/L)	1.9	(0.21, 1.4–2.6)	2.0	(0.21, 1.4–2.5)	1.9	(0.38, 1.1–2.6)	2.1	(0.27, 1.3–2.5)	<0.001
Cl^−^ (mmol/L)	102	(5.16, 87–113)	105	(7.87, 72–139)	106	(11.50, 78–138)	100	(9.73, 75–125)	<0.001
l-Lactate (enzymatic assay) (mmol/L), geo mean (95 % CI, range)	1.7	(1.6–1.9, 0.8–4.6)	3.7	(3.3–4.1, 0.8–14.4)	2.9	(2.2–3.8, 0.7–15.8)	1.9	(1.4–2.5, 0.3–11.5)	<0.001
Albumin (g/L)	30	(6.3, 16–46)	28	(5.8, 14–49)	28	(6.3, 18–41)	35	(6.2, 17–47)	<0.001
PO_4_^*3-*^ (mg/dl), geo mean (95 % CI, range)	0.7	(0.6–0.8, 0.1–3.0)	2.1	(1.8–2.3, 0.5–11.6)	3.5	(2.7–4.6, 1.0–12.9)	2.9	(2.5–3.3, 1.1–11.7)	<0.001
HCO_3_^−^ (mmol/L)	23.6	(3.4, 15.4–38.9)	19.0	(5.4, 3.5–35.8)	17.0	(6.1, 4.5–30.8)	24.2	(6.5, 3.9–41.3)	<0.001
pH	7.437	(0.053, 7.308–7.566)	7.398	(0.101, 6.931–7.615)	7.312	(0.182, 6.623–7.536)	7.442	(0.097, 7.054–7.549)	<0.001
pCO_2_ (kPa)	4.8	(0.92, 3.1–8.5)	4.2	(1.04, 2.2–8.1)	4.6	(2.10, 1.8–11.1)	4.8	(1.14, 1.9–7.2)	<0.001
SBD (mEq/L)	+1	(+3.6, −15 to +9)	+6	(+6.6, −11 to +29)	+9	(+7.6, +3 to +30)	0	(+7.6, −19 to +27)	<0.001
SID_eff_ (mEq/L)	33.5	(4.3, 22.9–50.8)	28.1	(6.1, 10.5–44.4)	26.8	(7.5, 11.4–44.2)	36.4	(6.9, 18.1–53.8)	<0.001
SID_app_ (mEq/L)	39.5	(4.4, 29.0–52.7)	36.3	(6.2, 13.8–52.4)	35.7	(7.2, 18.2–49.4)	43.0	(4.5, 32.1–53.3)	<0.001
SIG (mEq/L)	6.0	(3.7, −5.5 to 15.1)	8.2	(4.5, −2.4 to 23.4)	8.6	(7.7, −4.9 to 30.2)	6.6	(5.1, −2.4 to 24.1)	0.002

*Abbreviations: CI* confidence interval, *geo mean* geometric mean, *HCO*_*3*_^*−*^ bicarbonate, *pCO*_*2*_ partial pressure of carbon dioxide, *PO*_*4*_^*3-*^ phosphate, *SBD* standard base deficit; *SID*_*app*_ apparent strong ion difference, *SID*_*eff*_ effective strong ion difference, *SIG* strong ion gap

For all values, means (SD, range) are reported unless stated otherwise; geometric mean, 95 % CI and range are reported for non-normally distributed variables

*P values were determined by analysis of variance between the four participant groups. Variables with a normal or near-normal distribution were analysed directly; others were log-transformed toward normality, then analysed

### Identification of organic acids in plasma and urine

**Fig. 1:**
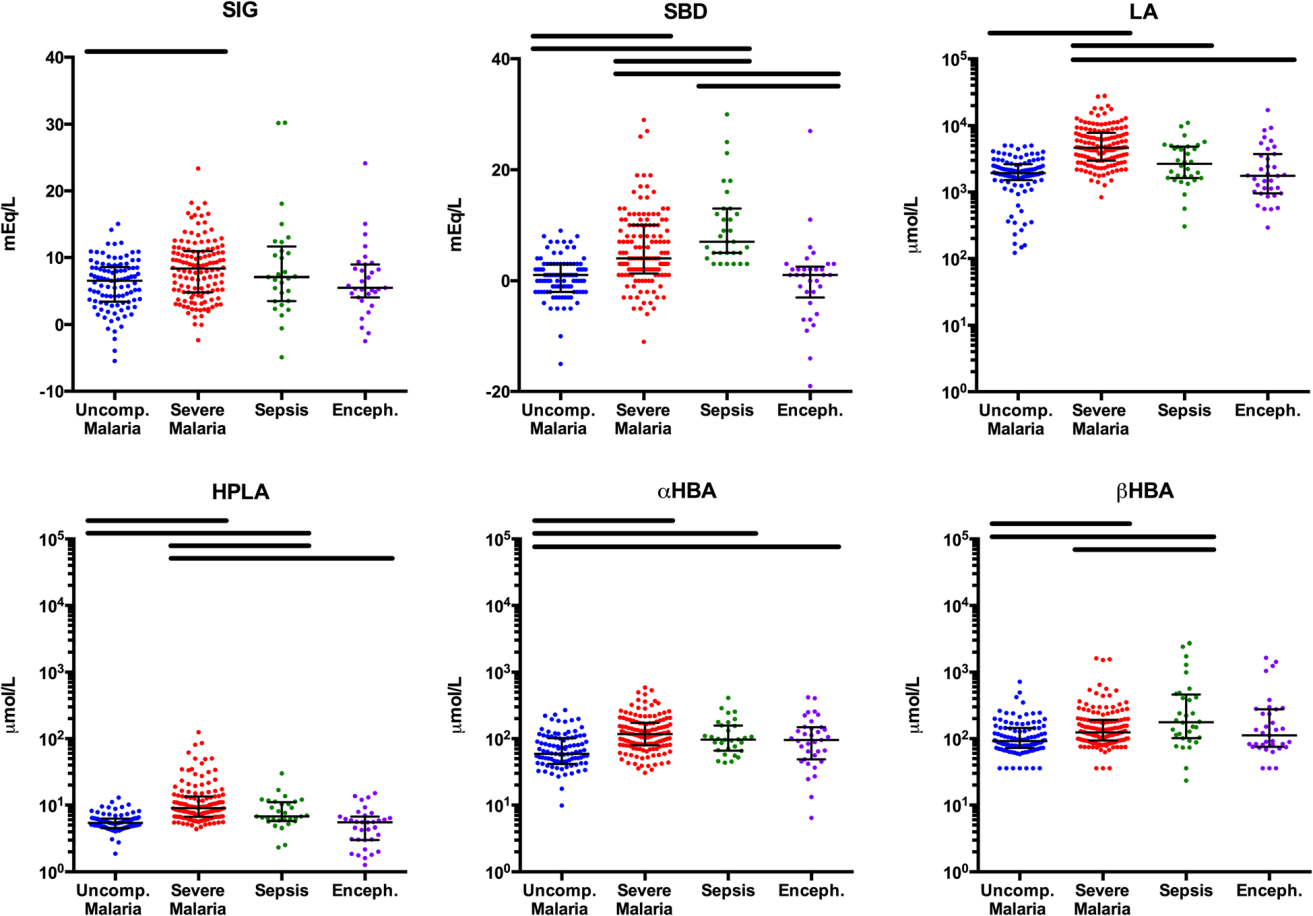
Plasma strong ion gap (SIG), standard base deficit (SBD) and concentrations of lactic acid (LA), hydroxyphenyllactic acid (HPLA), α-hydroxybutyric acid (α-HBA) and β-hydroxybutyric acid (β-HBA). *Black bars* above the scatterplots denote significantly different participant groups identified with Tukey’s test and *P*<0.05. *Error bars* represent medians and interquartile ranges

**Fig. 2:**
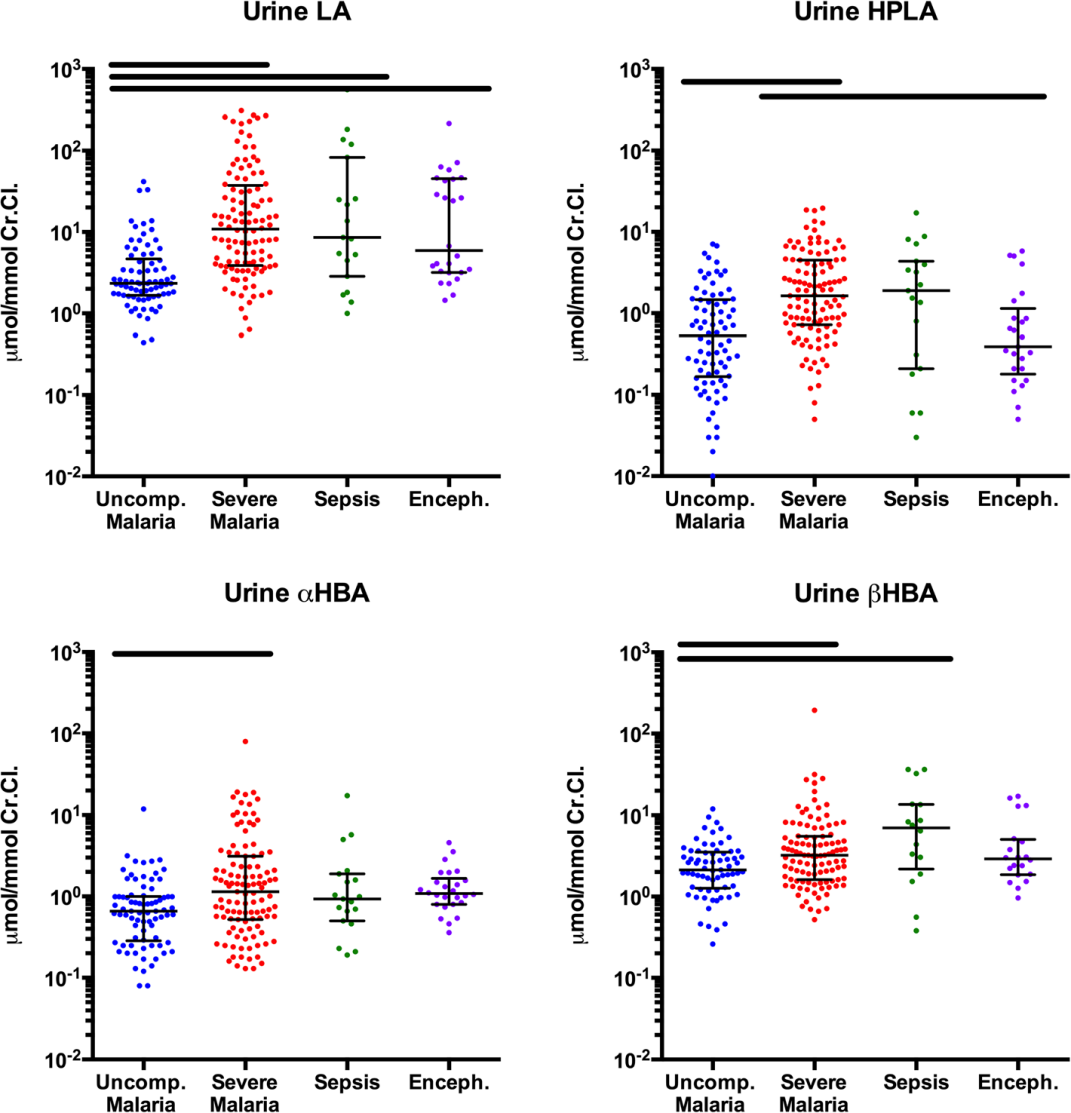
Urine concentrations of organic acids corrected for renal function. *Black bars* above the scatterplots denote significantly different participant groups identified with Tukey’s test and *P*<0.05. *Error bars* represent medians and interquartile ranges. *Abbreviations: CrCl* creatinine clearance, *LA* lactic acid, *HPLA* hydroxyphenyllactic acid, *α-HBA* α-hydroxybutyric acid, *β-HBA* β-hydroxybutyric acid

Of the eight candidate acids sought by LC-MS, four were present at detectable levels in both plasma and urine: LA, α-HBA, β-HBA and HPLA. EMA, MMA and α-KGA were undetectable in plasma but were detectable in urine. MA was undetectable in both plasma and urine. Table 3 summarises the observed range of acids detected in plasma and urine specimens taken from the study groups at the time of admission. Figures 1 and 2 compare plasma and corrected urine concentrations, respectively, between study groups.

**Table 3:** Quantification of organic acids in plasma and urine

Characteristics	Uncomplicated malaria N=102		Severe malaria N=138		Sepsis N=32		Encephalopathy N=35		*P* value*
	Geo mean	(95 % CI, range)	Geo mean	(95 % CI, range)	Geo mean	(95 % CI, range)	Geo mean	(95 % CI, range)	
Plasma concentration (μmol/L^a^)									
LA	1680	(1437–1965, 122–5019)	4912	(4389–5497, 835–27,958)	2615	(1970–3470, 305–11,002)	1898	(1380–2611, 293–17,132)	<0.001
HPLA	4.3	(3.7–5.0, UD–13.0)	10.6	(9.5–11.8, 4.4–124.7)	7.0	(5.4–9.0, UD–30.0)	4.4	(3.4–5.6, UD–15.2)	<0.001
α-HBA	46.0	(34.6–61.0, UD–270.1)	117.7	(106.3–130.4, 30.7–590.1)	88.4	(59.4–131.5, UD–411.3)	86.8	(63.8–118.1, 6.4–419.7)	<0.001
β-HBA	102.7	(91.9–114.8, UD–715.7)	143.9	(128.9–160.5, UD–1620.0)	223.5	(147.4–338.8, UD–2720.0)	146.8	103.9–207.3, UD–1651.0)	<0.001
Corrected urine concentrations (μmol/mmol CrCl)^a,b^		N=78		N=112		N=19		N=26	
LA	2.85	(2.32–3.49, 0.44–41.77)	12.97	(9.67–17.40, 0.54–1357.22)	13.47	(5.54–32.74, 1.00–555.30)	10.92	(6.05–19.72, 1.45–215.30)	<0.001
HPLA	0.48	(0.34–0.66, 0.01–7.08)	1.61	(1.28–2.04, 0.05–19.61)	1.15	(0.46–2.85, 0.03–17.12)	0.50	(0.29–0.88, UD–5.82)	<0.001
α-HBA	0.63	(0.51–0.78, 0.08–11.86)	1.26	(0.98–1.63, UD–79.63)	1.06	(0.61–1.84, 0.19–17.37)	1.16	(0.91–1.48, 0.36–4.57)	<0.001
β-HBA	2.11	(1.75–2.53, UD–11.99)	3.42	(2.83–4.12, UD–193.30)	5.52	(2.66–11.46, UD–36.28)	3.40	(2.27–5.09, UD–17.11)	<0.001
EMA	0.34	(0.27–0.43, 0.06–17.46)	0.26	(0.21–0.32, UD–9.58)	0.37	(0.0.24–0.57, UD–2.33)	0.36	(0.26–0.49, UD–2.25)	0.145
MMA	0.23	(0.19–0.28, 0.03–2.85)	0.20	(0.16–0.24, UD–2.97)	0.19	(0.13–0.27, UD–0.45)	0.21	(0.14–0.31, 0.05–4.75)	0.646
α-KGA	2.25	(1.72–2.95, UD–5.44)	3.18	(2.53–3.98, UD–27.11)	3.52	(1.82–6.82, UD–12.91)	3.89	(2.58–5.86, UD–26.41)	0.128

*Abbreviations: LA* lactic acid, *geo mean* geometric mean, *HPLA* hydroxyphenyllactic acid, *α-HBA* α-hydroxybutyric acid, *β-HBA* β-hydroxybutyric acid, *EMA* ethylmalonic acid, *MMA* methylmalonic acid, *α-KGA* α-ketoglutaric acid, *CrCl* creatinine clearance, *UD* undetectable

*Acid concentrations were log-transformed toward normality before analysis of variance. For plasma specimens, undetectable concentrations were treated as half the lower limit of detection to allow inclusion in intergroup comparisons. For corrected urine concentrations, undetectable values were treated as half the lowest value calculated from a detectable specimen

^a^For all plasma specimens, EMA, MMA, α-KGA and malonic acid (MA) were assayed but undetectable. For all urine specimens, MA was assayed but undetectable

^b^Corrected urine concentrations were adjusted for impaired creatinine clearance by incorporating the urine/plasma creatinine ratio as described in the Methods section

LA contributed most to the acid load in plasma and was significantly higher in severe malaria than in uncomplicated malaria, sepsis or encephalopathy.

There was a large difference in geometric mean (95 % CI) plasma HPLA concentration in patients with severe malaria (10.6 μmol/L; 9.5–11.8) compared with those with sepsis (7.0 μmol/L; 5.4–9.0), uncomplicated malaria (4.3 μmol/L; 3.7–5.0) or febrile encephalopathy (4.4 μmol/L; 3.4–5.6). Renal function–corrected urine concentrations of HPLA were also higher in patients with severe malaria than in those with uncomplicated malaria or encephalopathy.

Plasma β-HBA concentrations were significantly higher in patients with severe malaria than in those with uncomplicated malaria. Patients with sepsis had the highest relative and absolute concentrations of β-HBA in both plasma and urine. Levels of α-HBA in patients with severe malaria, sepsis or encephalopathy were higher than in those with uncomplicated malaria, but α-HBA levels were not distinguishable between patients with severe malaria and the reference groups. Corrected α-HBA urine concentrations were higher in patients with severe malaria than in patients with uncomplicated malaria.

Among participants with a positive SIG, the median total proportion of SIG that could be explained by HPLA, α-HBA and β-HBA was 3.4 % in patients with severe malaria (interquartile range [IQR] 2.1–7.0), compared with 4.2 % in patients with bacterial sepsis (IQR 2.0–15.6). The median proportion of SIG attributable to HPLA alone was 0.13 % in severe malaria (IQR 0.09–0.21), and 0.08 % in sepsis (IQR 0.04–0.18); these contributions were significantly different (*P*=0.018).

Plasma creatinine was correlated with HPLA (*r*=0.68; *P*<0.001) and β-HBA (*r*=0.21; *P*=0.016). Weaker correlations were seen between bilirubin and all four acids and between the parasite biomass marker *Pf*HRP2 and all four acids. GCS score correlated inversely with all four acids. The complete correlation matrix is provided in Additional file 1: Table S1.

### Prognostic significance of organic acids and their correlation with severity syndromes in patients with severe malaria

**Fig. 3:**
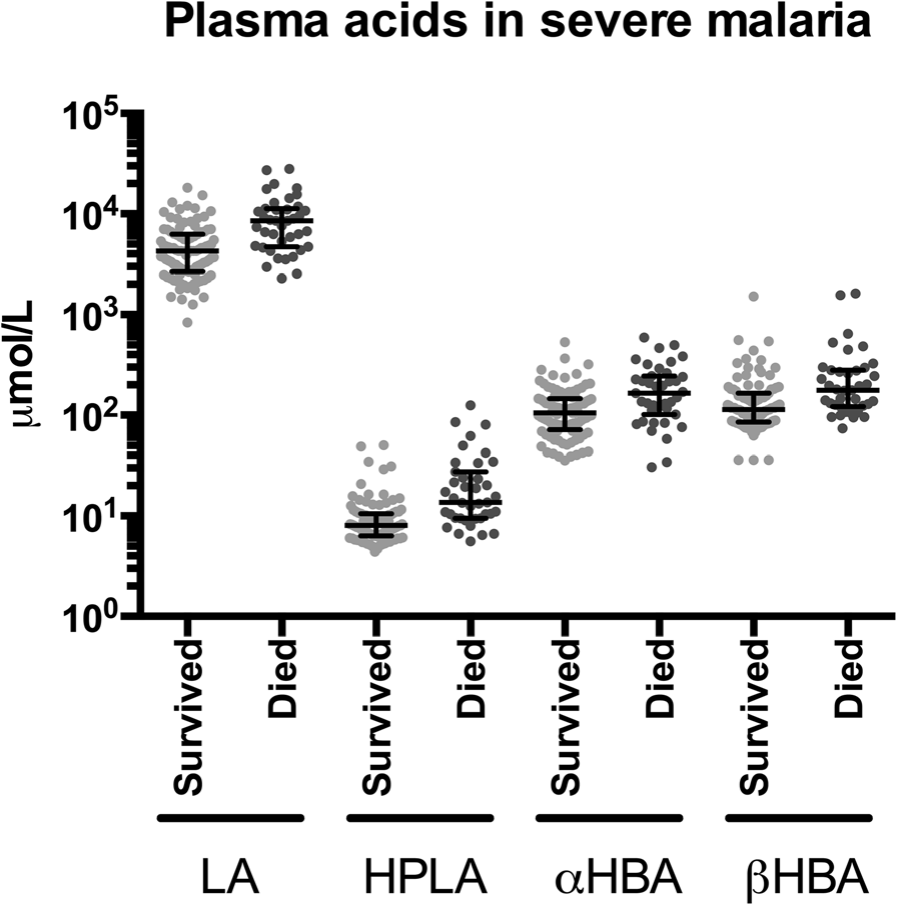
Plasma concentrations of organic acids in severe malaria, stratified by disease outcome. Comparisons are drawn between patients who survived to discharge (n=99) and those who died (n=39). For all four acids, plasma concentrations were significantly higher among patients who later died, by unpaired *t* test of log-transformed concentrations (*P*<0.001 for LA, HPLA and β-HBA; *P*=0.001 for α-HBA). *Error bars* represent medians and interquartile ranges. *Abbreviations: LA* lactic acid, *HPLA* hydroxyphenyllactic acid, *α-HBA* α-hydroxybutyric acid, *β-HBA* β-hydroxybutyric acid

Admission plasma concentrations of LA, HPLA, α-HBA and β-HBA were each higher in patients with severe malaria who later died than in those who survived (Fig. 3). Of the four detectable acids, HPLA had the highest prognostic utility with an AUROCC of 0.79 (95 % CI 0.70–0.87), followed by LA with an AUROCC of 0.77 (95 % CI 0.69–0.86). The AUROCC for SBD was 0.75 (95 % CI 0.65-0.85), and the AUROCC for pH was 0.74 (95 % CI 0.64–0.83). Urine LA and HPLA also had prognostic value, but less than that of their plasma concentrations (data not shown).

Logistic regression models were developed for patients with severe malaria, with in-hospital death as the dependent variable, to assess the prognostic value of plasma and urine organic acid concentrations (Table 4). An alternative multivariate model using clinical variables, including creatinine and *Pf*HRP2, was undertaken, but it did not improve the performance of the model (data not shown). In the model of plasma acids in 138 patients (39 of whom died), HPLA and LA had independent prognostic value, with the combined model providing an AUROCC of 0.81. Among 112 participants with available urine specimens (34 of whom died), LA was the only urinary acid of significant independent prognostic value. The AUROCC for a model based on urinary LA alone was 0.72.

**Table 4:** Summary of logistic regression models based on plasma acid concentrations (N=138) or urine acid concentrations (N=112) for probability of fatal outcome in participants with severe malaria

	Univariate analysis					Multivariate analysis				
	β	(95 % CI)	OR	(95 % CI)	*P*	β	(95 % CI)	OR	(95 % CI)	*P*
Regression of plasma acid concentrations										
LA	1.74	(1.00–2.47)	5.67	(2.72–11.86)	<0.001	1.24	(0.43–2.05)	3.45	(1.54–7.76)	0.003
HPLA	1.76	(1.02–2.51)	5.82	(2.76–12.26)	<0.001	1.25	(0.48–2.02)	3.48	(1.61–7.52)	0.001
α-HBA	1.22	(0.53–1.91)	3.40	(1.71–6.78)	0.001	–		–		
β-HBA	1.09	(0.45–1.73)	2.98	(1.57–5.64)	0.001	–		–		
Regression of urine acid concentrations										
LA	0.54	(0.25–0.84)	1.723	(1.28–2.31)	<0.001	0.54	(0.25– 0.84)	1.72	(1.28–2.31)	<0.001
HPLA	0.58	(0.21–0.96)	1.791	(1.23–2.61)	0.002	–		–		
α-HBA	0.23	(−0.06 to +0.52)	1.259	(0.94–1.68)	0.118	–		–		
β-HBA	0.38	(0.02–0.74)	1.459	(1.02–2.09)	0.039	–		–		
MMA	−0.03	(−0.36 to +0.29)	0.966	(0.70– 1.34)	0.836	–		–		
EMA	0.12	(−0.22 to +0.46)	1.126	(0.80–1.58)	0.489	–		–		
α-KGA	−0.13	(−0.43 to +0.16)	0.877	(0.65–1.18)	0.382	–		–		

*Abbreviations: OR* odds ratio, *LA* lactic acid, *HPLA* hydroxyphenyllactic acid, *α-HBA* α-hydroxybutyric acid, *β-HBA* β-hydroxybutyric acid, *EMA* ethylmalonic acid, *MMA* methylmalonic acid, *α-KGA* α-ketoglutaric acid, *AUROCC* area under the receiver operating characteristic curve

Goodness of fit of the plasma acid model: AUROCC=0.81; Hosmer-Lemeshow=0.94; *P*=0.612, N=138. Goodness of fit of the urine acid model: AUROCC=0.72; Hosmer-Lemeshow=0.72; *P*=0.459, N=112. Concentrations were log-transformed to normality before analysis. All variables in univariate analysis were initially included in the model, then backward stepwise selection was performed, removing variables on the basis of *P*≥0.05

## Discussion

Metabolic acidosis with an increased SIG and high SBD is a prominent feature of severe falciparum malaria, and severity of the acidosis is closely linked to mortality [4, 16, 23]. The present study shows that organic acids other than lactate also have a strong prognostic significance for death in adult patients with severe malaria. HPLA, α-HBA and β-HBA were all present at higher concentrations in plasma and urine of patients with severe malaria than in those with uncomplicated disease and also at higher plasma concentrations in patients who died than in those who survived. This identifies HPLA as an acid with strong independent prognostic significance, despite low absolute concentration. The elevation of HPLA was more pronounced in severe malaria than in patients with severe sepsis, whereas SBD and SIG were elevated in both groups. Patients with febrile encephalopathy were not acidotic, but some showed a strongly elevated SIG, suggesting a potential pathophysiological role for organic acids; however, the profile of acids we describe in severe malaria is distinct from that of non-malarial encephalopathy [24].

LA was detectable in plasma at a considerably higher concentrations than any other organic acid and was a strong independent predictor of death in patients with severe malaria, with a higher AUROCC than reported previously [4]. We consistently observed higher concentrations of LA detected by LC-MS than we did with the conventional enzymatic assay. This may reflect the fact that conventional enzymatic assays detect only the l-lactate isomers, whereas LC-MS detects both l- and d-isomers. Human metabolic pathways produce only l-lactate, but both isomers are produced by *P. falciparum*, as the parasite relies heavily on incomplete oxidation of glucose into LA for its own energy supplies [25]. Bacterially derived d-lactate can accumulate in the context of multiorgan failure, with increased transposition of bacteria from the ischaemic gastrointestinal tract [26–28], and it is noteworthy that d-lactate can cause an encephalopathy in humans ranging from disorientation to coma [29]. The hypothesis that d-lactate could contribute to the pathogenesis of severe malaria requires further quantification and study.

HPLA was identified at higher concentrations in the plasma and urine of participants with severe malaria than in reference groups, and it was a strong and independent predictor of mortality in patients with severe malaria. Although total HPLA concentrations were relatively low, the high prognostic value of HPLA may reflect perturbations in associated metabolic pathways, with related acids also contributing to the total acid load. Alternatively, this may indicate strong biological activity of the anion at low concentrations or local accumulation in affected tissues. HPLA is a product of l-tyrosine catabolism [30]. Plasma and urine HPLA concentrations are very low in health, but higher levels have been described in the context of critical illness [18, 31–35]. Adults with post-operative sepsis or hepatic encephalopathy have increased plasma and urine concentrations [34, 36]. Urinary excretion of HPLA has also been described in patients with tyrosinaemia type III, a rare inborn error of metabolism [37]. In these patients with neurological impairment, 4-hydroxyphenylpyruvate dioxygenase, the enzyme that converts hydroxyphenylpyruvate (HPPA) to homogentisate, is deficient, causing diversion of HPPA to HPLA. This raises the possibility that increased HPLA levels in severe malaria could be related to a functional blockade in the downstream pathway of HPPA. Interestingly, phenylalanine—an indirect precursor of HPPA—is elevated in both paediatric and adult patients with severe malaria [38, 39]. In addition to human metabolic pathways, gastrointestinal microflora (including *Enterococcus faecalis*, *Escherichia coli*, bifidobacteria and lactobacilli) can be a source of HPLA [35]. High levels of parasite sequestration occur in the gastrointestinal vasculature in severe malaria [40]. This could drive local ischaemia and bacterial translocation, with higher levels of HPLA entering the circulation. HPLA accumulation may also reflect changes to routes of clearance in severe disease. Its strongest identified biochemical correlation is with creatinine, so it may accumulate disproportionately in patients with kidney injury. It is noteworthy that other phenolic acids have been shown to influence brain metabolism in experimental studies in vivo and in vitro, altering neuronal and glial metabolism and inducing coma in rats [41–43]. A potential neurobiological role of HPLA in severe and cerebral malaria remains to be further elucidated.

β-HBA was high in plasma and urine of patients with severe malaria but highest in patients with severe sepsis. Concentrations were not independently correlated with mortality. β-HBA is synthesised in the liver from free fatty acids, and increased production of ketoacids can be related to the augmented metabolic demand, coupled with reduced oral caloric intake, in severe illness [18, 44, 45]. Elevated plasma β-HBA has been associated with severe malaria and other severe illnesses among children in Kenya [44].

Concentrations of α-HBA were high in patients with severe malaria, sepsis or encephalopathy, but they were not an independent predictor of mortality. α-HBA is a product of threonine and methionine catabolism, but it is also produced in glutathione synthesis during the conversion of cystathionine to cysteine. The glutathione pathway is upregulated to counteract oxidative stress, which is increased during severe malaria [22, 46]. Raised α-HBA levels may thus reflect increased production of glutathione. Raised levels of α-HBA have been found in the urine of patients with lactic acidosis, which may be related to the redox state of the cell [47].

We acknowledge the limitations of this hypothesis-generating study and the need for further, targeted exploration of the mechanisms by which the identified acids influence the pathogenesis of malaria. The heterogeneity of organ dysfunction in both severe malaria and severe sepsis complicates our understanding of both conditions, particularly with regard to the metabolic pathways implicated. This heterogeneity reduces the statistical power of our analysis; yet, significant conclusions emerge.

## Conclusions

Concentrations of HPLA were significantly higher in patients with severe malaria than in patients with sepsis, febrile encephalopathy or uncomplicated malaria, which suggests a disease-specific role. HPLA also has prognostic significance independent of LA. Further research into the kinetics and biological effects of these acids, as well as into the contributing metabolic pathways, is warranted.

## Key messages

- Previously unmeasured organic acids could be identified in patients with severe falciparum malaria using a novel targeted LC-MS approach.
- A tyrosine metabolite and two ketoacids were detected at increased concentrations in patients with severe malaria.
- Lactate and 4-hydroxyphenyllactic acid were predictive of mortality in patients with severe malaria.
- Our findings highlight metabolic pathways potentially implicated in disease pathogenesis.

## Additional file

Additional file 1: Table S1. AUROCCs of biochemical and clinical markers with prognostic significance at the time of initial assessment of patients with severe malaria as predictors of death, and correlation matrix of biochemical and clinical markers. (DOC 43 kb)

## Abbreviations

ANOVA: analysis of variance
AUROCC: area under the receiver operating characteristic curve
CI: confidence interval
CrCl: creatinine clearance EDTA, ethylenediaminetetraacetic acid
EMA: ethylmalonic acid
GCS: Glasgow Coma Scale
Hb: haemoglobin
HCO_3_^−^: bicarbonate
HPLA: hydroxyphenyllactic acid
HPPA: hydroxyphenylpyruvate
IQR: interquartile range
LA: lactic acid
LC-MS: liquid chromatography-mass spectrometry
MA: malonic acid
MMA: methylmalonic acid
OR: odds ratio
pCO_2_: partial pressure of carbon dioxide
*Pf*HRP2: *Plasmodium falciparum* histidine-rich protein 2
SBD: standard base deficit
SBP: systolic blood pressure
SD: standard deviation
SID: strong ion difference
SIG: strong ion gap
UD: undetectable
WHO: World Health Organisation
α-HBA: α-hydroxybutyric acid
α-KGA: α-ketoglutaric acid
β-HBA: β-hydroxybutyric acid
