# Erratum to: the role of previously unmeasured organic acids in the pathogenesis of severe malaria

**DOI:** 10.1186/s13054-015-1116-1

**Published:** 2015-11-02

**Authors:** M. Trent Herdman, Natthida Sriboonvorakul, Stije J. Leopold, Sam Douthwaite, Sanjib Mohanty, M. Mahtab Uddin Hassan, Richard J. Maude, Hugh W. F. Kingston, Katherine Plewes, Prakaykaew Charunwatthana, Kamolrat Silamut, Charles J. Woodrow, Aniruddha Ghose, Kesinee Chotinavich, Md Amir Hossain, M. Abul Faiz, Saroj Mishra, Natchanun Leepipatpiboon, Nicholas J. White, Nicholas P. J. Day, Joel Tarning, Arjen M. Dondorp

**Affiliations:** Mahidol-Oxford Tropical Medicine Research Unit, Faculty of Tropical Medicine, Mahidol University, Bangkok, Thailand; Department of Chemistry, Faculty of Science, Chulalongkorn University, Bangkok, Thailand; Department of Clinical Tropical Medicine, Faculty of Tropical Medicine, Mahidol University, Bangkok, Thailand; Ispat General Hospital, Rourkela, Orissa India; Chittagong Medical College Hospital, Chittagong, Bangladesh; Centre for Tropical Medicine and Global Health, Nuffield Department of Medicine, University of Oxford, Oxford, UK; Global Health Division, Menzies School of Health Research, Darwin, Australia; Dev Care Foundation, Dhaka, Bangladesh

After publication of the original article [1] it was found that author Aniruddha Ghose was accidentally omitted from the list of authors. This has now been corrected with this erratum. The updated ‘Authors’ Contributions’ section reads as follows:

MTH, NS, and SJL drafted the manuscript. NS, NL, and JT devised and performed LCMS analyses. NJW, NPJD, and AMD conceived of the study. SD, MTH, RJM, MAF, MMUH, MAH, SMo, SMi, CJW, and AG contributed to the design. MTH, SJL, SD, RJM, HWFK, KP, PC, KS, and KC provided clinical and diagnostic data collection and critically revised the manuscript. All authors read and approved the final manuscript.
