# Supplementary material for: The role of previously unmeasured organic acids in the pathogenesis of severe malaria
Source: Crit Care. 2015 Sep 7;19(1):317. doi: 10.1186/s13054-015-1023-5 (PMC4561438; doi:10.1186/s13054-015-1023-5)
Supplement: Additional file 1: Table S1. — AUROCCs of biochemical and clinical markers with prognostic significance at the time of initial assessment of patients with severe malaria as predictors of death, and correlation matrix of biochemical and clinical markers. (DOC 43 kb) [file 13054_2015_1023_MOESM1_ESM.doc]

**Additional file 1**

**Supplementary Material Online:**

**Table S1. AUROCCs of biochemical and clinical markers with prognostic significance at the time of initial assessment of patients with severe malaria as predictors of death, and correlation matrix of biochemical and clinical markers.**

|  | **Prognostic Significance** | **Correlation**  **Matrix** |  |  |  |  |  |  |  |  |
| --- | --- | --- | --- | --- | --- | --- | --- | --- | --- | --- |
|  | **AUROCC (95% CI)**  As predictor of death from severe malaria | **Plasma HPLA**  r  P  N | **Plasma αHBA**  r  P  N | **Plasma βHBA**  r  P  N | **SBD**  r  P  N | **pH**  r  P  N | **Creatinine**  r  P  N | **Bilirubin**  r  P  N | ***Pf*HRP2**  r  P  N | **GCS**  r  P  N |
| **Plasma LA** | 0.77 (0.69-0.86) | 0.50  <0.001  138 | 0.66  <0.001  138 | 0.53  <0.001  135 | 0.56  <0.001  136 | -0.46  <0.001  136 | 0.14  0.095  135 | 0.27  0.002  135 | 0.37  <0.001  126 | -0.23  0.007  138 |
| **Plasma HPLA** | 0.79 (0.70-0.87) | … | 0.38  <0.001  138 | 0.43  <0.001  135 | 0.67  <0.001  136 | -0.58  <0.001  136 | 0.68  <0.001  135 | 0.46  <0.001  135 | 0.43  <0.001  126 | -0.22  0.011  138 |
| **Plasma αHBA** | 0.70 (0.59-0.80) | … | … | 0.80  <0.001  135 | 0.39  <0.001  136 | -0.23  0.008  136 | 0.07  0.415  135 | 0.20  0.019  135 | 0.37  <0.001  126 | -0.23  0.008  135 |
| **Plasma βHBA** | 0.71 (0.62-0.80) | … | … | … | 0.41  <0.001  133 | -0.35  <0.001  133 | 0.21  0.016  132 | 0.35  <0.001  132 | 0.30  0.001  123 | -0.19  0.027  135 |
| **SBD** | 0.75 (0.65-0.85) | … | … | … | … | 0.74  <0.001  136 | 0.61  <0.001  133 | 0.41  <0.001  125 | 0.42  <0.001  133 | -0.24  0.006  136 |
| **pH** | 0.74 (0.64-0.83) | … | … | … | … | … | -0.49  <0.001  133 | -0.37  <0.001  133 | -0.31  0.001  125 | 0.08  0.366  136 |
| **Creatinine** | 0.62 (0.51-0.72) | … | … | … | … | … | … | 0.44  <0.001  135 | 0.36  <0.001  125 | 0.03  0.709  135 |
| **Bilirubin** | 0.67 (0.56-0.78) | … | … | … | … | … | … | … | 0.21  0.018  125 | -0.04  0.675  135 |
| ***Pf*HRP2** | 0.66 (0.55-0.77) | … | … | … | … | … | … | … | … | -0.27  0.002  126 |
| **GCS** | 0.74 (0.65-0.83) | … | … | … | … | … | … | … | … | … |

Parametric assessment for normal distribution variables (or variables log-transformed toward normality); non-parametric assessment for GCS, which lacked a normal distribution.

Abbreviations: AUROCC, area under the receiver operating characteristic curve; HPLA, hydroxyphenyllactic acid; αHBA, α-hydroxybutyric acid; βHBA, β-hydroxybutyric acid; SBD, standard base deficit; *Pf*HRP2, *Plasmodium falciparum* histidine-rich protein 2; GCS, Glasgow coma scale; r, Pearson r; P, two-tailed P-value; N, number of subjects compared; LA, lactic acid.
